# Usefulness of component resolved analysis of cat allergy in routine clinical practice

**DOI:** 10.1186/s13223-016-0163-8

**Published:** 2016-11-15

**Authors:** Katharina Eder, Sven Becker, Marion San Nicoló, Alexander Berghaus, Moritz Gröger

**Affiliations:** 1 Department of Oto-Rhino-Laryngology, Head & Neck Surgery, Ludwig-Maximilians-University, Marchioninistr. 15, 81377 Munich, Germany; 2 Department of Oto-Rhino-Laryngology, Head & Neck Surgery, Johannes-Gutenberg-University, Mainz, Germany

**Keywords:** Cat allergy, Native cat extract diagnostic, Fel d 1, Fel d 2, Fel d 4

## Abstract

**Background:**

Cat allergy is of great importance, and its prevalence is increasing worldwide. Cat allergens and house dust mite allergens represent the major indoor allergens; however, they are ubiquitous. Cat sensitization and allergy are known risk factors for rhinitis, bronchial hyperreactivity and asthma. Thus, the diagnosis of sensitization to cats is important for any allergist.

**Methods:**

70 patients with positive skin prick tests for cats were retrospectively compared regarding their skin prick test results, as well as their specific immunoglobulin E antibody profiles with regard to their responses to the native cat extract, rFel d 1, nFel d 2 and rFel d 4. 35 patients were allergic to cats, as determined by positive anamnesis and/or nasal provocation with cat allergens, and 35 patients exhibited clinically non-relevant sensitization, as indicated by negative anamnesis and/or a negative nasal allergen challenge.

**Results:**

Native cat extract serology testing detected 100% of patients who were allergic to cats but missed eight patients who showed sensitization in the skin prick test and did not have allergic symptoms. The median values of the skin prick test, as well as those of the specific immunoglobulin E antibodies against the native cat extract, were significantly higher for allergic patients than for patients with clinically non-relevant sensitization. Component based diagnostic testing to rFel d 1 was not as reliable. Sensitization to nFel d 2 and rFel d 4 was seen only in individual patients.

**Conclusion:**

Extract based diagnostic methods for identifying cat allergy and sensitization, such as the skin prick test and native cat extract serology, remain crucial in routine clinical practice. In our study, component based diagnostic testing could not replace these methods with regard to the detection of sensitization to cats and differentiation between allergy and sensitization without clinical relevance. However, component resolved allergy diagnostic tools have individual implications, and future studies may facilitate a better understanding of its use and subsequently may improve the clinical management of allergic patients.

## Background

There are numerous reasons why sensitization to cats plays an important role in the clinical routine of an allergist. First, the prevalence of cat allergy is increasing [1–3]. In 2009, a study was published describing sensitization to cats in 26% of adults in Europe [4], whereas in 1992, 8.8% of adults were sensitized to cats [5]. In German children, sensitization to cats is reported in 9.6% of boys and 6.6% of girls [6]. Generally, the prevalence of sensitization is dependent on age. It increases throughout childhood and peaks during adolescence [7–10]. Sensitization to cats is associated with increased bronchial hyperresponsiveness in adults and children [11–14]. Additionally, cat allergy is a major risk factor for developing asthma and rhinitis [15, 16]. Cat allergens are found in households with cats but are also considered ubiquitous. Exposure can occur in schools, occupational environments and outdoors [17]. The amount of cat allergen in schools correlates directly with the number of school children with cats in their homes [18]. In homes without a cat, sensitization can occur if a sufficient number of households in the community own a cat. Additionally, pet ownership differs among countries, e.g., 15% of German households have cats, whereas in Greece only 4% of households, and in the Netherlands 27% own cats [19].

Therefore, the diagnosis of sensitization to cats is important, irrespective of cat ownership. There is a trend toward costly component resolved analysis, which has been described as the beginning of new era in the diagnosis of allergies [20, 21], compared to standard diagnostic approaches, such as the extract based SPT (skin prick test) and serology against native extract. These standard tools are more cost effective because the extract usually contains all of the allergenic components within one test; however, the increased regulatory demands within the European Union for any allergen solution used for diagnostic tests, such as SPT or provocation tests, have driven the use of component resolved analysis [22].

Konradsen et al. provided a detailed overview of the potential of molecular based diagnostic methods in diagnosing allergies to furry animals [23]. Briefly, dander is considered the primary source of cat allergens [24, 25]. The major cat allergen is Fel d 1, a uteroglobin, which was purified by Ohman et al. in 1974 [26]. It is produced in the skin and salivary glands [24, 27, 28] and is the sensitizing allergen in up to 95% [29–32]. The characterization of many other allergens followed this one. The most important are serum albumin Fel d 2 [33, 34], which is important for cross-reactivity to serum albumins of other animals, and lipocalin Fel d 4 [35], which cross-reacts with lipocalin allergens from other animals [12, 36]. Further cat allergens have also been characterized, such as cystatin Fel d 3 [37], cat IgA Fel d 5 and IgM Fel d 6 [38], lipocalin Fel d 7 and latherin-like Fel d 8 [39].

The aim of the study was to investigate whether cat allergen component analysis can replace or add anything to the performance of extract based SPT and serology in routine clinical practice.

## Methods

### Patient data

The allergy database of the Department of Oto-Rhino-Laryngology, Head and Neck Surgery of the Ludwig-Maximilians-University in Munich consists of patient information stated in the case history and diagnostic results. The database was retrospectively scanned for patients presenting to our department between 2011 and 2015 with possible cat allergy or sensitization. Within these 5 years, a total of 1202 patients underwent comprehensive allergy diagnostic testing, and 413 (34%) were sensitized against cats. Only a small set of these data records were complete with regard to the relevant parameters of our study. 70 patients with proven allergy (n = 35) or clinically silent sensitization (n = 35) were selected. The selection criteria for the allergy group were as follows: a positive SPT to cats and positive anamnesis for cat allergy and/or a positive nasal allergen challenge. For the sensitization group, the criteria were as follows: a positive SPT to cats and negative anamnesis for cat allergy and/or a negative nasal challenge. The exclusion criteria were a combination of an SPT to cats of I and a CAP class of 0 for the specific antibody to native cat extract using the FEIA (Fluorescence Enzyme Immunoassay) method (UniCAP-FEIA, Thermo Fisher Scientific, Freiburg, Germany). These patients were rated as not sensitized to cats.

### Skin prick test (SPT)

The SPT solution for cats by ALK-Abelló, Wedel, Germany was used. The SPT was considered positive with a wheal >3 mm in diameter (I = ≥3 to <4, II = ≥4 to <5, III = ≥5 to <6, IV = ≥6) in combination with Histamine dihydrochloride solution at 1 mg/ml as positive control and allergen-free saline solution as negative control. It was read 20 min after application. The procedure was in line with published guidelines [40–43].

### Fluorescence enzyme immunoassay (FEIA)

IgE reactivity to purified natural allergen extract and allergen components was measured using the FEIA method (UniCAP-FEIA, Thermo Fisher Scientific, Freiburg, Germany) with a commercially available test kit (Thermo Fisher Scientific, Freiburg, Germany), in accordance with the instructions of the manufacturer. Total IgE and specific IgE antibodies to native cat extract and to the allergen components rFel d 1, nFel d 2, and rFel d 4 were measured. In addition, in patients with positive specific IgE towards serum albumin nFel d 2 and lipocalin rFel d 4, specific IgE to rCan f 2, rCan f 1, nCan f 3 and rEqu c 1 were determined, respectively. The results are given in concentration units (kU/l). The positive cutoff value was >0.35 kU/l as suggested by Thermo Fisher Scientific (Freiburg, Germany).

### Nasal provocation test

The intranasal challenge test solution for cats designated for nasal provocation was glycerin-free and had an allergen concentration of 100,000 SQ-E/ml (ALK-Abelló, Wedel, Germany). The intranasal challenge was performed in accordance with the current guidelines [44]. Testing was considered positive if rhinomanometry was decreased >40% at 150 Pa on the side tested with the allergen, as well as for a symptom score >3 or a decrease in rhinomanometry >20% in combination with a symptom score >2. The symptom score consists of registered secretion, irritation and remote symptoms [45]. The nasal provocation test was used to differentiate between allergy and sensitization without clinical relevance in selected patients.

### Statistical analyses

Statistical analyses and the graphical presentation were performed on a Lenovo Thinkpad X61 s with SigmaPlot (Jandel Corp., San Rafael, CA, USA) and Excel (Microsoft, Redmond, WA, USA). All of the data failed normality testing (Shapiro–Wilk). Therefore, we used the median for descriptive statistics and the Mann–Whitney Rank Sum Test for testing statistically significant differences in the median values between the two groups. For the graphical presentation of the native cat extract results, the data are presented as the median with the 75th percentile as the error bar. Correlation between native cat extract and rFel d 1 results was calculated by Spearman Rank Order Correlation. A value of *p* < 0.05 was considered significant.

## Results

We compared two groups of 35 patients each, one with cat allergy and the other with sensitization to cat allergens without clinical relevance. Patient data is summarized in Table 1.

**Table 1:** Patient demographics and characteristics

	Sensitization	Allergy
	(n = 35)	(n = 35)
Male	18 (51%)	15 (43%)
Female	17 (49%)	20 (57%)
Age (range 8–86 years)	35	33
Mono-sensitized	1 (3%)	2 (6%)
Oligo-sensitized	15 (43%)	17 (49%)
Poly-sensitized	19 (54%)	16 (46%)
Asthma	7 (20%)	18 (51%)
Food allergy	9 (26%)	14 (40%)

Values are number of patients total and percent of each evaluated group

Age is given as a mean

Briefly, both groups were comparable with regard to their gender distribution, age and sensitization profiles. We distinguished between patients, mono-sensitized to cat, patients that were oligo-sensitized to 1–2 additional perennial or seasonal allergens, and patients, poly-sensitized to 3 or more allergens in addition to cat. Neither group showed any statistically significant differences in total IgE antibody concentrations. The allergy group had a median total IgE antibody concentration of 188 kU/l, and the sensitization group had a median concentration of 228 kU/l. 36% of all patients reported asthma (18 (51%) in the cat allergy group and 7 (20%) in the sensitization group). An allergic reaction to food, mainly Bet v 1-homologous PR10-protein containing food, was reported by 33% of patients [14 (40%) in the allergy group and 9 (26%) in the sensitization group]. Allergic symptoms with meat were not reported.

**Fig. 1:**
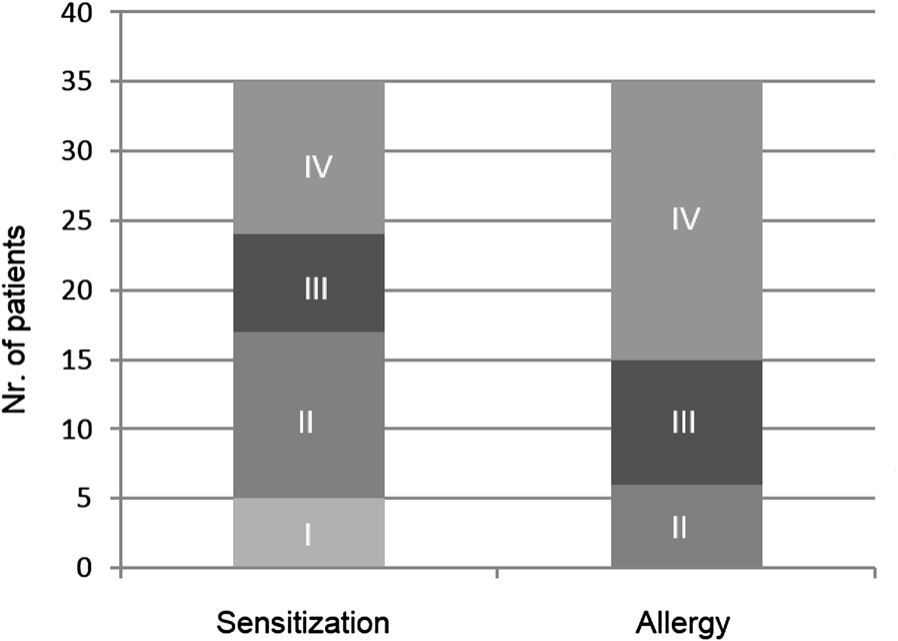
Results of SPT. Levels of SPT reactivity (*I*–*IV*) in patients with clinically silent sensitization (n = 35) and allergy (n = 35) to cat. The median in the allergy group is higher than in the sensitization group (*p* = 0.004)

We compared different diagnostic tools within the allergy and sensitization groups. Figure 1 shows the distribution of the SPT results in both groups. The mean values of the SPT were III (range I–IV) in the sensitization group and IV (range II–IV) in the allergy group. This difference was statistically significant (*p* = 0.004).

Table 2 shows the sensitization profiles to different cat components of allergic and asymptomatic patients.

**Table 2:** Sensitization profile to different cat components

	Sensitization	Allergy
	(n = 35)	(n = 35)
Native cat positive	27 (77%)	35 (100%)
IgE level (kU/l)	1.58 (0.02–82.4)	2.95 (0.5–47.5)
rFel d 1 positive	26 (74%)	32 (91%)
IgE level (kU/l)	1.08 (0.02–53.9)	2.59 (0.18–20.4)
nFel d 2 positive	1 (3%)	2 (6%)
IgE level (kU/l)	0.00 (0–15.5)	0.01 (0–5.66)
rFel d 4 positive	5 (14%)	6 (17%)
IgE level (kU/l)	0.03 (0–36.5)	0.03 (0–12.9)

Values are number of patients total and percent of each evaluated group

IgE levels are given as median and range

**Fig. 2:**
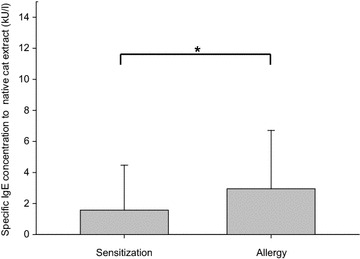
Specific IgE concentrations to native cat extract. Median specific IgE concentrations in response to native cat extract (in kU/l) in patients with clinically silent sensitization and allergy to cat (*p = 0.012), with the 75th percentile as the *error bar*

**Fig. 3:**
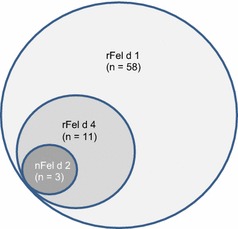
Prevalence of specific IgE antibodies toward rFel d 4 and n Fel d 2. The prevalence of specific IgE antibodies toward rFel d 4 and n Fel d 2 are low. Out of all 70 patients 58 had specific IgE antibodies toward r Fel d 1, 11 of these were also positive for r Fel d 4. Of these, 3 patients also had specific IgE antibodies against nFel d 2

Prevalence of a specific IgE against native cat extract was 100% in the allergy group and 77% in the sensitization group. The 8 patients (23%) who were negative for the native extract in the sensitization group had different SPT results. 3 of those patients had an SPT of II, 3 patients had an SPT of III and 2 patients had an SPT of IV. The prevalences of rFel d 1, nFel d 2 and rFel d 4 were 91, 6, and 17% in the allergy group and 74, 3 and 14% in the sensitization group. Comparing the 2 groups, we found a statistically significant difference in the median of the specific IgE antibody concentration toward the native cat extract. The median in the allergy group was 2.95 kU/l (range 0.5–47.5 kU/l), whereas the median in the sensitization group was 1.58 kU/l (range 0.02–82.4 kU/l) (*p* = 0.012) (Fig. 2). Comparing the median of the specific IgE antibodies against rFel d 1, we did not find a statistically significant difference between the groups. The median in the allergy group was 2.59 kU/l (range 0.18–20.4 kU/l), and in the sensitization group, it was 1.08 kU/l (range 0.02–53.9 kU/l) (*p* = 0.07). 93% of patients having specific IgE against rFel d 1 also had specific IgE towards native cat extract, whereas only one patient having specific IgE against rFel d 1 did not have specific IgE towards native cat extract. This patient was not allergic to cat. The correlation between rFel d 1 and native cat extract was highly significant (coefficient = 0.896 with *p* < 0.001). The low prevalences of specific IgE antibodies toward nFel d 2 and rFel d 4 did not allow any assumptions regarding differentiation between allergy and silent sensitization with respect to these parameters. Figure 3 illustrates the distribution of all cat components analysed: out of 58 patients having specific IgE against rFel d 1, 11 also had IgE towards rFel d 4. Out of these 11 patients, 3 patients also had specific IgE to nFel d 2 (Fig. 3). 6 of 11 patients (55%, 3 allergic, 3 asymptomatic) with specific IgE antibodies to rFel d 4 reported asthma. 2 of these asthmatic patients also had specific IgE antibodies to nFel d 2.

Regarding cross-reactivity between serum albumins and lipocalins, additional specific IgE antibody testing revealed that out of 70 patients, all 3 patients tested positive for IgE antibodies toward nFel d 2, cat serum albumin, also had specific IgE antibodies toward nCan f 3, dog serum albumin. 1 of these patients had documented subjective allergic symptoms to dogs as well as to cats. 11 of 70 patients had specific IgE antibodies toward lipocalin rFel d 4. Eight of these patients were also positive for IgE antibodies against horse lipocalin rEqu c 1, although only two of these patients documented subjective allergic symptoms to horse, and only one of them also reported clinical symptoms when exposed to cats. Four of the 11 patients who tested positive for rFel d 4 also had IgE antibodies against dog lipocalin rCan f 1. Two of these patients were also sensitized to dog lipocalin rCan f 2. Three of these patients had documented subjective allergic symptoms when exposed to dogs and cats. None of our patients reported allergic symptoms with pork or meat.

## Discussion

This study examines whether component resolved diagnostic testing with rFel d 1, nFel d 2 and rFel d 4 in cat allergy can replace or add anything to the performance of extract based diagnostic analysis with SPT and serology against native cat extract in routine clinical practice.

The SPT is highly sensitive for detecting sensitization to native cat allergens. Patients in both groups were selected with a positive SPT. Pesonen et al. showed that 100% of children with positive SPT results also tested positive later in life [46]. Nevertheless, there are cases, such as children, pre-medicated patients or dermatographic patients, where the SPT is not the primary test of choice. Specific IgE antibodies to native cat extract also have relatively high sensitivity. In our study, 89% of patients had specific IgE antibodies to native cat extract. However, only 77% of the sensitized group had IgE antibodies to native cat extract, whereas in the allergy group, specific IgE antibodies to native cat extract were present in all patients. Consequently, in our study, native extract serology missed one sensitized patient without clinical symptoms but was able to reliably detect sensitization in an allergic patient. In addition, in our study, the level of SPT positivity, as well as the concentration of specific IgE toward native cat extract, may indicate the existence of an allergy to cats. Allergic patients had a significantly higher result in both extract based diagnostic approaches compared to sensitized patients without clinical symptoms.

The component resolved diagnostic tests in our study were less sensitive. The prevalences of specific antibodies for the allergen components rFel d 1, nFel d 2 and rFel d 4 were 83, 4 and 16% taking both groups together. 91% of patients who were allergic to cats reacted to rFel d 1 in our study. Thus, 9% of allergic patients would have not been detected as being sensitized to cats with component resolved diagnostic testing alone. In the literature, up to 95% of patients with cat allergies have specific IgE antibodies to rFel d 1 [26, 29–32]. In our study, the concentrations of specific IgE antibodies against rFel d 1 were higher in the allergy group than in the sensitization group, but the difference was not statistically significant. Therefore, we could not conclude that rFel d 1 diagnostic analysis is as specific as extract based testing with regard to differentiation between cat allergy and clinically irrelevant sensitization. However, we could not exclude a type 2 error. Also, nasal challenge to cat allergen was not performed in all patients. In routine clinical practice, especially in poly-sensitized patients, provocation testing cannot be performed to test all sensitizations with regard to their clinical relevance. If anamnesis of cat allergy is evident, patients do not necessarily undergo provocation testing. In our study, 16 patients obtained provocation testing due to vague anamnesis. However, we could not eliminate inaccuracy by false information or interpretation of symptoms by the patient.

In the literature, the prevalence of reactivity against cat serum albumin in sensitized patients, independent of clinical relevance, is described as being between 15 and 25% [32, 47, 48]. Spitzauer et al. even describe a prevalence of up to 30% among cat and dog allergic patients with respect to reactivity against the serum albumin of cats and dogs [49]. The prevalence of a positive IgE reaction to serum albumin nFel d 2 in our allergic patients was 6%. The prevalence of specific IgE antibody concentrations against lipocalin rFel d 4 in the literature is also much higher than that found in our study. In our study, it was 16% for all patients taken together. Smith et al. report levels of up to 63% in sensitized patients, although the concentration levels in this study were very low [35]. In our study, all patients with positive IgE levels against nFel d 2 and rFel d 4 were also positive for native cat extract and rFel d 1. Sensitization to multiple components was seen in six allergic as well as in five asymptomatic patients, therefore, sensitization to multiple components did not indicate the likelihood of being allergic to cat. However, the number of patients also being sensitized to nFel d 2 and rFel d 4 were very low.

In summary, in our study, component based diagnostic analysis could not replace standard extract based methods with regard to detecting sensitization to cats or to differentiate between allergy and asymptomatic sensitization in sensitized patients. The following question remains: what do component resolved diagnostic tests add to standard extract based methods?

Konradsen et al. recently published a review comprehensively addressing this question [23]. They stated the importance of identifying specific allergen components associated with asthma and severe disease. Bjerg et al. reported a higher prevalence of asthma in children co-sensitized to rFel d1 and rFel d 4 than in children sensitized to r Fel d 1 alone [50]. We also saw a higher prevalence of reported asthma (55%) in patients with IgE reactivity, not only toward rFel d 1, but also toward nFel d 2 and/or rFel d 4. On the other hand, only a few asthmatic patients were tested positive for nFel d 2 and/or rFel d 4. Therefore, testing of these 2 components alone could not reliably identify asthmatic patients. Wisniewski et al. described an association between high levels of IgE antibodies to nFel d 2 and rFel d 4 and atopic dermatitis in cat-allergic children [51]. In our allergic collective, 33% of patients with IgE levels against nFel d 2 and/or rFel d 4 self-reported atopic dermatitis. In addition, the above mentioned authors note the importance of identifying clinically relevant sensitizations to cross-reactive molecules and those associated with allergic syndromes. IgE antibodies to nFel d 2, as well as to rFel d 4, are cross-reactive to serum albumins and lipocalins of other animals. Spitzauer et al. showed that a high percentage of patients sensitized to serum albumin react to cat and dog [49]. This was confirmed in our study; all patients with IgE antibodies reactive to cat serum albumin nFel d 2 also reacted to dog serum albumin nCan f 3. Regarding the lipocalins, the sequence identities are usually much lower than for serum albumins. This explains the low co-prevalence of sensitization to cat lipocalins and dog lipocalins in our study. Only a few lipocalins, such as Fel d 4 and Equ c 1, show much higher sequence identities of approximately 60% [36, 52]. The cross-reactivity between the two serum albumins in our study was high, as well. 8 (73%) of 11 patients having IgE antibodies reactive to rFel d 4 also reacted to rEqu c 1. Liccardi et al. stressed the importance of component based diagnostics in detecting allergic sensitization to common pets, such as cats and dogs, and their potential usefulness in predicting the risk of allergic sensitization to other less common animals as it has been suggested that cross-reacting mechanisms may play a role in this process, especially in the absence of any possible direct or indirect contact. They argue the use of this approach for the detection of major risk factors for severe respiratory disease among sensitized patients without prior animal exposure, especially before acquiring a new pet or initiating contact with a pet for work or leisure [53]. IgE cross-reactions within the serum albumin and lipocalin families, their implications for the diagnosis of allergies and their clinical relevance are the subject of many research studies. Konradsen et al. concluded that there is clear evidence for the clinical importance of component resolved diagnostic testing in specific cases and also defined several topics that should be addressed in future to broaden the spectrum of the utility of these tests in routine clinical practice with regard to sensitization to furry animals [23].

In conclusion, extract based methods, such as the SPT and serology to native cat extract, are crucial in routine clinical practice for detecting sensitization to cats and cannot be replaced by a single allergen component or a combination of allergen components, and therefore remain the most effective tool for diagnosing sensitization to cats. Moreover, in our study, results of SPT and specific IgE antibodies against native cat extract were significantly higher in patients allergic to cats compared to patients without clinical symptoms. This trend could not be seen for the allergen components rFel d 1, nFel d 2 and rFel d 4.

## Abbreviations

SPT: skin prick test
IgE: immunoglobulin E
FEIA: fluorescence enzyme immunoassay
